# Development and Pilot of an Interactive Online Course on Antimicrobial Stewardship in Companion Animals

**DOI:** 10.3390/antibiotics10050610

**Published:** 2021-05-20

**Authors:** Nonke E. M. Hopman, Jaap A. Wagenaar, Ingeborg M. van Geijlswijk, Els M. Broens

**Affiliations:** 1 Department Biomolecular Health Sciences, Faculty of Veterinary Medicine, Utrecht University, Utrecht, Yalelaan 1, 3584 CL Utrecht, The Netherlands; J.Wagenaar@uu.nl (J.A.W.); E.M.Broens@uu.nl (E.M.B.); 2 Wageningen Bioveterinary Research, Houtribweg 39, 8221 RA Lelystad, The Netherlands; 3 IRAS Veterinary Pharmacology and Therapeutics Group, Department Population Health Sciences, Faculty of Veterinary Medicine, Utrecht University, Yalelaan 106, 3584 CM Utrecht, The Netherlands; i.m.vangeijlswijk@uu.nl

**Keywords:** antimicrobial stewardship, companion animals, small private online course (SPOC), optimizing antimicrobial prescribing, veterinary medicine

## Abstract

A holistic approach to antimicrobial use (AMU) and prescribing is needed to combat the problem of antimicrobial resistance (AMR). Previously, an antimicrobial stewardship programme (ASP) was developed, introduced, and evaluated in 44 Dutch companion animal clinics, which resulted in an optimization of AMU. As a follow-up to this, an online course was developed to promote awareness of AMU, AMR, and responsible antimicrobial prescribing. The aim of this paper is to describe the development and pilot, including evaluation, of this course, which will be disseminated more widely among Dutch companion animal veterinarians. The interactive programme consists of a major e-learning component and two online, face-to-face meetings. The course comprises five different parts corresponding with five consecutive weeks. Theory on several topics is offered, for example on AMU and AMR in general, Dutch regulations and guidelines on veterinary AMU, behavioural change, and possible methods to quantify AMU. Additionally, several assignments are offered, for example to reflect upon one’s own current antimicrobial prescribing behaviour. Interactive discussion and peer-to-peer learning are promoted. Since September 2020, the course has been offered in a pilot phase, and the feedback is promising. Evaluation of the pilot phase will result in recommendations for further optimization and dissemination.

## 1. Introduction

Effective antimicrobials are essential for current and future human medicine. Animal health and welfare rely on effective antimicrobials, too. As antimicrobial resistance (AMR) is a threat to global health and development, multisectorial action is needed to address this issue, since AMR does not adhere to species or environmental boundaries [1,2,3,4]. An important driver for the emergence and increase in AMR is antimicrobial use (AMU) [5]. In general, three main strategies are proposed to save antimicrobials as non-renewable resources for the future [6,7]: (1) Infection prevention and control to prevent the spread of (resistant) bacteria, since every infection prevented is one that needs no treatment; (2) stimulating research and development of new (classes of) antimicrobials (AMs) and other treatment options; (3) securing the efficacy of the existing antimicrobial treatment options, including ensuring access.

Antimicrobial stewardship mainly focuses on the third strategy. In human healthcare, antimicrobial stewardship programmes (ASPs) generally refer to specific programmes or series of interventions to monitor and optimize AMU at the hospital or primary care level [8,9,10]. These ASPs have been developed and implemented for decades and have proven to be effective in optimizing AMU and reducing AMR levels [8,11]. The primary goal of antimicrobial stewardship is to achieve optimum clinical outcomes and ensure cost-effectiveness of therapy, while keeping the unintended consequences of AMU to a minimum, including toxic effects, selection of pathogenic organisms, and the emergence of AMR [8,12]. Antimicrobial stewardship is advocated to improve the quality of AMU [8]. In companion animals, AMs are frequently prescribed, driving selection for resistance [13,14,15,16,17,18]. Of particular concern is the veterinary use of critically important antimicrobials for human medicine, especially those considered highest priority critically important antimicrobials (HPCIAs) [19,20,21]. At the same time, companion animals live close to humans providing opportunities for interspecies transmission of (resistant) microorganisms [22,23,24,25,26,27]. In veterinary medicine, the introduction of antimicrobial stewardship is a rather new phenomenon. It usually encompasses numerous elements of improved AMU (e.g., increasing awareness of (inter)national guidelines on veterinary AMU, use of diagnostic microbiology, and use of alternatives to AMs), and it is often associated with country-wide surveillance of AMU and development of (inter)national guidelines on AMU [28]. Little research has been published that evaluates the effectiveness of interventions seeking to optimize antimicrobial prescribing in companion animals [29]. Recently, Singleton et al. [21] performed a trial to assess the effect of education, in-depth benchmarking, and follow-up meetings on the prescribing of HPCIAs. This trial revealed effective strategies for reducing the prescribing of these HPCIAs. In 2015–2018, a fairly extensive ASP was developed, introduced, and evaluated in 44 Dutch companion animal clinics (the Antimicrobial Stewardship and Pets (ASAP) project), which resulted in an optimization of AMU in these veterinary clinics [30]. Based on the successes of this ASAP project, an online course was developed to raise awareness on AMU and AMR and promote responsible antimicrobial prescribing. The objective of this paper is to describe the development and pilot, including evaluation, of this course, which will be disseminated more widely among Dutch companion animal veterinarians.

## 2. Results/Final Online Course

The final, interactive programme consists of a major e-learning component and two online, face-to-face meetings with participating veterinarians and two course organizers. Resources used for the development of the e-learning content and the online meetings include peer-reviewed scientific publications, Dutch regulations and guidelines on veterinary antimicrobial use, expertise from the course organizers, and parts of the former ASAP project. The course organizers have a background in veterinary medicine, veterinary microbiology, hospital and veterinary pharmacy, clinical pharmacology and epidemiology. The target audience of the online course is Dutch companion animal veterinarians. Prior to the pilot, the e-learning module was evaluated by a small group of volunteers to gather feedback on possible technical issues and course content.

### 2.1. Content

The online course comprises five different parts. Each part is intended to take one week, and so the total time to complete the online course is five consecutive weeks. The expected time investment for the participating veterinarians is 2–3 h per week. The online course is offered to 10–15 veterinarians simultaneously within a course group, to facilitate interactive discussions and openness during the course.

For a detailed overview of the course content for every single week/subject, see Table 1. In summary, week 1 starts with a general introduction to AMR and why it is considered important. This introduction is aimed at increasing autonomous motivation regarding the topic and creating awareness of AMR. The main part of week 1 consists of individual assignments to reflect on one’s own current AM prescribing behaviour (what?) and on factors influencing one’s own AM prescribing behaviour (why?). Week 2 offers knowledge and theory about AMU, AMR, and current Dutch regulations and guidelines on veterinary AMU. It also offers knowledge about antimicrobial stewardship and diagnostic workups, including the use of bacterial culturing and susceptibility testing. Week 3 offers theory on behavioural change and communication, thereby introducing the RESET model, together with practical examples and tools to improve AM prescribing behaviour, and it finishes with an individual assignment on behaviour. Week 4 offers theory on possible general quantification methods of veterinary antimicrobial use. Options to quantify antimicrobial use within their own companion animal clinic are also introduced, and possible barriers for quantification and monitoring of AMU in veterinary clinics are touched upon. In addition, EU Regulation 2019/6 is introduced, which requires a standardized way of organizing and collecting AMU in all companion animal clinics [31]. In Week 5, the final week, participants are asked to formulate five different SMART (specific, measurable, achievable, realistic and time-related) action points they will start to work on in their own clinic and how they plan to do this. During the entire course, participants are encouraged to discuss and learn from each other on the online platform (see Section 2.3). During and after the course, participants can ask antimicrobial stewardship-related questions by email to the course organizers.

### 2.2. Online Meetings

Part of the programme is based on two online meetings in course weeks 3 and 5. The aim of these interactive meetings is to promote peer-to-peer learning and discussion both between the participants themselves and between participants and course organizers, answer participants’ questions, and elaborate in more detail on subjects introduced or questions raised during the e-learning. To overcome distance and time restrictions, it was decided to offer these meetings as online meetings (Microsoft Teams, Version 1.4.0.2781). In week 3, the online meeting is hosted by the veterinary microbiologist and the project leader. In week 5, the online meeting is hosted by the hospital pharmacist-clinical pharmacologist and the project leader. Both semi-structured meetings are scheduled for 1 to 1.5 h, and are prepared by the project leader. Discussions during the meetings are based on questions asked by participants beforehand on the online course forum, by email, or ones they ask during the meeting.

### 2.3. Online Platform and Dissemination

The course is accessible via the Lifelong Learning (LLL) Platform of Utrecht University. This interactive platform offers the opportunity to post questions during the entire course and have structured online discussions on every topic. Course organizers (i.e., the project leader, hospital pharmacist-clinical pharmacologist, and veterinary microbiologist) check the discussion forums on the platform two to three times per week to identify issues at an early stage, answer questions, and keep an eye on the ongoing discussions.

The course is advertised on the website of Utrecht University, Faculty of Veterinary Medicine, via newsletters from Utrecht University, Faculty of Veterinary Medicine, and via websites and newsletters from Dutch veterinary associations. Participation is offered for free during this initial pilot phase of the project. Currently, professional and continuing education are not obligatory for Dutch companion animal veterinarians. However, an increasing number of organizations and clinics strongly advise this. Participation in the online course is accredited for veterinary professional and continuing education, and one hour of time invested is equivalent to one post-educational credit. Therefore, veterinarians are assigned 15 post-educational credits after participation in the entire course.

### 2.4. Pilot Phase and Evaluation Process

Participants can express their interest in participating in the online course to the project leader verbally or by email. Veterinarians are asked to provide their name and a person-specific email address in order to participate. Other personal information, such as working place or age, is not collected. After that, any questions are answered, course groups are formed, and login information to access the online platform is provided. To date (in April 2021), approximately 87 veterinarians have started the course, and 84 of them have already completed it. Veterinarians from throughout the Netherlands, including those parts that are less centrally situated, are participating in the course.

Once they have completed the course, veterinarians are asked to fill out a short course evaluation questionnaire. Forty (48%) veterinarians have done this. The results of the questionnaire on the achievement of the learning objectives are shown in Table 2. In short, participating veterinarians report being more aware of the importance of AMR and responsible AMU, they mention being more aware of their own AM prescribing behaviour, and they state that they are now using AMs more responsibly. Besides, all respondents state that they appreciated participating in this online course (totally agree = 27, agree = 11, tend to agree = 2). Also, all forty respondents agreed that time-investment and effort required to participate were optimal (totally agree = 1, agree = 23, tend to agree = 16). The two interactive, online meetings were especially highly appreciated, as well as the assignments to reflect on their own AM prescribing behaviour (what and why? Week 1) and the theoretical parts on AMU, AMR, and antimicrobial stewardship (Week 2). The main unfavourable comments concern the chosen online platform, which has some serious technical drawbacks. Another issue regularly mentioned during the evaluation is the fact that theory and good stewardship practices are not always practically applicable in daily practice, where many factors influence antimicrobial prescribing. Further, some veterinarians stated that they already prescribe antimicrobials responsibly, and so the course affirmed their behaviour. However, these veterinarians reported less responsible AM prescribing by veterinarians in other clinics. This suggests the need for those veterinarians to participate in a course on antimicrobial stewardship, but it also raises the question as to how those veterinarians can be reached. For this pilot, quite an effort had to be made to recruit the current number of participating veterinarians; some veterinarians were very enthusiastic about participating, but others had to be enthused.

## 3. Discussion

The development and pilot of an interactive, online course based upon a previously introduced but extensive antimicrobial stewardship intervention programme to optimize awareness on AMU and AMR and improve antimicrobial prescribing among Dutch companion animal veterinarians was described. This online course aims to combine the benefits of time- and distance-independent learning with interactive and peer-to-peer learning.

Good education has been recognized as one of the cornerstones for supporting and effecting a response to the challenge of AMR [28,32,33]. Theoretical aspects and knowledge are therefore offered in all parts of the e-learning. Initially, a common awareness of the importance of AMU and AMR is created. Subsequently, antimicrobial stewardship and the cues of the RESET model are addressed. Measuring is knowing, which is pivotal in assessing AMU, monitoring changes over time, and enabling the benchmarking of AMU. Therefore, the course continues with possible quantification methods of AMU, both in humans and animals [34,35,36,37].

Studies on AMU in companion animals generally demonstrate a high use of critically important AMs for human medicine [13,14,15,16,37,38,39,40]. However, standardized quantitative data on systemic AMU in companion animals are scarce, and an obligatory and standardized European policy requiring countries to report their veterinary AMU for companion animals is currently lacking, but will be in place by 2030 [31,41]. Insight into topical AMU in companion animals is even more scarce [34,37]. However, a current lack of a standardized approach to quantify all AMU in companion animals should not form a barrier for introducing antimicrobial stewardship in individual companion animal clinics. Quantitative and qualitative assessment of antimicrobial prescribing in (individual) companion animal clinics is necessary for optimizing AMU. A first step towards a better registration and reporting of AMU is reflecting upon what information is currently available and, equally important, what information is missing in individual companion animal clinics. Monitoring AMU over time within one’s own clinic requires a standardized approach, with the same measures used as numerator and denominator. For instance, Méndez and Moreno [41] and Gomez-Poveda and Moreno [40] offer examples of how the number of treatments and the number of dogs treated can be assessed quite easily both quantitatively and qualitatively by looking at individual patient records. This could, for example, be done for 24 randomly selected patients (dogs, cats, and rabbits) per individual clinic by taking one random patient for every letter of the alphabet and then reviewing the patient records, both qualitatively and quantitatively, for a given period of one year. Additional options to reflect on AMU within one’s own veterinary clinic are discussed during the online meetings, and related questions are also asked by email after the course has been completed.

A massive open online course (MOOC) format and a small private online course (SPOC) format were considered for the current online course. In the former ASAP project, every participating clinic followed its own, individual programme, combined with two interactive, face-to-face meetings with participating veterinarians from other clinics and the course organizers, and one individual face-to-face feedback meeting. Interactive meetings and discussions with other veterinarians and course organizers, as well as individual, clinic-based feedback, were highly appreciated. Participants from the former ASAP project reported that they were interested in seeing how their prescribing compared to other veterinarians (benchmarking), which is also underlined by other studies [42]. Having interactive discussions with other participants and course organizers might deepen insight and motivate participants to consider alternative attitudes. Therefore, interaction with peers and discussion was supported and a more private (SPOC) course format was preferred.

Besides, interactive discussion also provides valuable information for the course organizers on issues regarding AMU that veterinarians face during daily practice, which could lead to new research or follow-up topics. The issue most frequently raised during the present online course as being very difficult in everyday practice was determining the optimal treatment duration. Veterinarians experience challenges in determining the optimal course duration, because evidence-based information is missing and, as a result, current guidelines on veterinary AMU do not address course duration for many clinical indications. Questions such as ‘Can I treat an animal with AMs for too short a period and could that cause AMR? Does it harm to treat too long with AMs?’ and ‘How can I determine beforehand how long an animal should ideally be treated for with AMs?’ were questions encountered in every course group of participating veterinarians. More evidence-based research is warranted to fill knowledge gaps on the optimal duration and to evaluate the outcome of different treatment regimens for different indications [43,44]. Meanwhile, clear tools should be offered to veterinarians to deal with these knowledge gaps, starting with implementing structural therapy evaluation. Other information sources could also be used, like existing veterinary literature, recommendations from veterinary experts, recommendations in the specific product characteristics, and if needed, recommendations from research in human medicine, although this should be done with caution [45]. During the online course, the importance of therapy evaluation was emphasized, so that the effect of a chosen therapy could be monitored and therapy suspension, curtailment, or prolongation could be considered. Another issue that the participants often raised as challenging was perioperative antimicrobial use. The participants mentioned many different approaches to when and how antimicrobials were used perioperatively, and which types of antimicrobials were chosen. More research is needed to develop evidence-based standard practices for perioperative AMU as well.

Evaluation of the pilot phase will be used to make recommendations for further improvement and dissemination. The evaluation will also be used to enable possible future implementation in the Master of Veterinary Medicine of Utrecht University curriculum. Time investments from both the organizers in the development and implementation of the course as well as the time invested by participants need to be evaluated. The development phase of the present course comprised approximately half a year. The online face-to-face interactive parts received a positive evaluation, but these parts also require active time investments by the course organizers at set times every time an online course is organized (2 × 1.5 h). The introduction of this online course during the current COVID-19 situation could be seen as an opportunity, as it can be followed flexibly in terms of time and location. However, Dutch companion animal veterinarians are currently experiencing a higher workload, e.g., due to quarantine restrictions or illness of colleagues combined with more consultations due to the increase in the number of pets during the lockdown period, which means they have less time to participate in courses. This may have restricted participation in this antimicrobial stewardship e-learning programme, as some veterinarians stated this as a reason for not participating. This is supported by the findings of Tompson et al. [29], indicating that financial reimbursement or provision of veterinary staff to cover clinical duties could be crucial in supporting the completion of antimicrobial stewardship activities.

The online platform chosen appeared to be a critical issue and needs reconsideration and/or adaptations. The technical drawbacks encountered need to be addressed, since they could be a barrier for participants to participate and to be actively involved in online discussions. Besides, it remains to be seen whether participation in this online course indeed promotes awareness of AMU and AMR and improves antimicrobial prescribing in the long-term among Dutch companion animal veterinarians. Antimicrobial use as an outcome measure is not currently part of the course evaluation since measuring and monitoring AMU in companion animal clinics is a very labour-intensive process due to the lack of a central and uniform registration system of AMU in these clinics [30]. In the future, national systems will be developed and implemented to comply with the new EU regulation 2019/6 that obliges EU Member States to report not just sales but also use data of antimicrobial veterinary medicinal products. By 2029, use data in companion animals will also need to be collected. However, the feedback and evaluation from participating veterinarians is promising and encouraging, and eventually individual practices will also be able to extract and interpret these data from their practice management systems.

Furthermore, since current e-learning on antimicrobial stewardship is non-obligatory, participants with less autonomous motivation on this subject are and will not be reached. Previous research has shown that autonomous motivation (in contrast with controlled motivation) might be a facilitator for medical residents to participate in a non-obligatory e-learning on antibiotic prescribing [46]. Some participating veterinarians indeed mentioned that they already try to adhere to Dutch legislation and guidelines on veterinary antimicrobial use. Participation in this e-learning has strengthened their knowledge and may stimulate them to enthuse other colleagues. However, other veterinarians who are not yet adhering to the guidelines and are possibly not interested in participating in a course on antimicrobial stewardship will be hard to reach. Possible options to enhance autonomous motivation, and with that to optimize e-learning efficiency, could be applied in the future, such as face-to-face education preceding the e-learning or statements by veterinary associations on the importance of responsible veterinary AMU. Another possible option to improve autonomous motivation is positive reactions and social pressure from peers, veterinary colleagues or veterinary associations. However, as long as post-educational training in general, and more specifically on optimizing AMU, is not mandatory, then not all veterinarians will be reached.

An annual follow-up or a refresher course could be useful for the long-term embedding of thoughts on antimicrobial stewardship and to update participants on best practices and guidelines on veterinary AMU, which evolve over time. Some participants in the present online course have stated their interest in a follow-up or update course. However, this will require further time investment, and so new financial resources will need to be found.

The current online course is based on the current Dutch situation concerning veterinary AMU and Dutch regulations and guidelines on veterinary AMU. Regulations, guidelines and target levels for appropriate AMU might differ per country [29,47,48,49]. Therefore, the exact content of the course cannot be copied to other countries. However, the general layout based on reflection, current AM prescribing behaviour, and offering several tools to improve AMU, combined with interactive discussions, is applicable in every country, as long as country-specific guidelines and regulations are introduced as well.

## 4. Materials and Methods/Programme Development

### 4.1. Previous Antimicrobial Stewardship and Pets Project (ASAP)

During the aforementioned ASAP project, an antimicrobial stewardship programme (ASP) to optimize AMU in Dutch companion animal clinics was developed. This was based on qualitative research [50] and field experiences from the co-authors involved in both human and veterinary medicine [30]. Cues from the RESET model to change human behaviour were also applied [51,52]. A support team (S Team) was assembled that was analogous to the human antibiotic stewardship teams (A teams) (www.ateams.nl (last accessed on 19 May 2021)), which included a veterinary microbiologist, a veterinary specialist in internal medicine of companion animals, a veterinary pharmacologist, a hospital pharmacist, and a project leader. The S Team members were involved in the entire project.

The separate intervention elements offered during this ASP comprised:

(1) Filling in patient evaluation forms per individual participating clinic.
(2) Two post-educational training evenings on AMR, (inter)national regulations and guidelines on responsible AMU, behavioural change and communication skills towards companion animal owners.
(3) Exercises to reflect on antimicrobial prescribing habits and guidelines within their own clinic.
(4) Signing a commitment form within their own clinic to use AMs responsibly.
(5) Benchmarking of quantitative AMU data with approximately 10 other participating veterinary clinics from the same region.
(6) Information leaflet for companion animal owners on responsible AMU and AMR.
(7) Asking questions to the S team members about AMU and AMR via email or phone.
(8) Feedback meeting—Every participating clinic was visited once by the project leader and one or two of the other S Team members to reflect on their clinic-specific AMU and prescribing strategies and habits. Veterinarians participated in this ASP voluntarily, albeit with financial reimbursement for participating veterinarians for their invested time. Participation in this multifaceted ASP to optimize AMU in companion animal clinics showed a positive effect on AMU in these Dutch companion animal clinics [30]. After participation, participants reported being more aware of their own antimicrobial prescribing behaviour and could all mention specific changes they had made in their own clinic. However, participation in the entire ASP was considered time-consuming by both the participants and the organizers. Therefore, a more widespread roll out of this intensive ASP was considered unfeasible.

### 4.2. Topics

To enable a wider reach to more Dutch companion animal veterinarians, the most feasible and effective elements of the ASP were selected based on an online questionnaire (adapted from Hulscher et al. and Kreuwel et al. [53,54]) sent to the participating clinics to evaluate participation in the ASP. This online survey was completed by 65 participants (both veterinarians and veterinary technicians) from the 44 clinics that participated in the ASAP project. Participation in the entire ASP was evaluated as most useful and supportive for optimizing AMU. The individual intervention elements of signing the commitment form, the possibility to ask ad-hoc questions to the S Team members, and the information leaflet for companion animal owners were evaluated as least useful and supportive for optimizing AMU. Individual intervention elements based upon reflection on one’s own antimicrobial prescribing behaviour and antimicrobial prescribing habits within one’s own clinic, benchmarking of AMU data, theory and knowledge about AMU, AMR, and current guidelines and regulations, and clinic specific feedback were evaluated as most useful and supportive. Therefore, these elements were selected for inclusion in the follow-up course, along with the cues from the RESET model to change human behaviour.

### 4.3. E-Learning

E-learning could offer a solution to the problem of time and distance limitations for both participants and course organizers, as experienced during the previous ASAP project. Once an e-learning course has been developed, participants can participate with a relatively small investment of time, the course is flexible, and there is no need to travel [46]. Use of e-learning in human medicine was shown to be effective in optimizing AMU [55,56] and in instilling knowledge, attitudes, and skills associated with safe and effective prescribing of medicines [57]. An online course was also recently introduced to improve the use of metagenomics in AMR surveillance [58]. Since peer-to-peer learning and discussions with other veterinarians and with course organizers were highly appreciated during the previous ASAP project, interactive elements to promote discussions between participating veterinarians and interactive contact moments with the course organizers were both incorporated.

### 4.4. Evaluation Process

Veterinarians were asked to fill out a short evaluation questionnaire after finishing the course to enable future improvements and possible further dissemination of the course. Completing the online questionnaire was estimated to take no more than 10 min. The questions (6-point Likert scale questions) were related to achievement of the learning objectives, fulfilment of prior expectations about the course, personal experiences, and changes in antimicrobial prescribing behaviour made during or after participation in the course. Participants agreed to data from this questionnaire being used anonymously to evaluate the course.

### 4.5. Ethics Approval

As no animal experiments are involved, the study was exempt from ethical approval. The final e-learning course encourages companion animal veterinarians to improve their antimicrobial prescribing according to current regulations and guidelines on veterinary AMU. Participating veterinarians remain fully autonomous in their daily practice.

## 5. Conclusions

Since September 2020, a multifaceted, interactive online course to optimize antimicrobial use in Dutch companion animals has been offered to Dutch companion animal veterinarians. Evaluation of the pilot phase resulted in recommendations for improvement and further dissemination of the course.

## Figures and Tables

**Table 1:** Detailed description of the online course content.

Week/Subject	Topic(s)	Learning Objective(s)	Elements
1 Introduction	Effective antimicrobials are essential for both human and animal health and welfare	To create awareness of AMR and on the importance of effective AMs.	Introductory video clip; Individual assignment based upon a dermatology case; Individual assignment on own prescribing behaviour with regard to general clinical cases; Theory on factors influencing prescribing behaviour; Individual assignment on factors influencing own prescribing behaviour; Peer feedback and discussion on individual assignments.
1 Current AM prescribing behaviour	Reflection on current AM prescribing behaviour	To gain insight into and to reflect on current AM prescribing behaviour: what do I do and why?	
2 Knowledge and Theory	Antimicrobial resistance	To increase knowledge of AMR and Antimicrobial Stewardship.	Theory with interactive questions and individual assignments on clinical cases and on the use of bacterial cultures and susceptibility tests; Background literature; Individual assignment on AMU within their own veterinary clinic; Individual assignment on a dermatology case; Individual assignment on ‘what to do when antimicrobials are not indicated?’; Individual assignment on therapy evaluation; Peer feedback and discussion on individual assignments.
	Antimicrobial use	To promote the use of current guidelines on veterinary AMU.	
	Antimicrobial Stewardship		
	Current Dutch regulations and guidelines on veterinary AMU		
	Use of bacterial cultures and susceptibility testing		
	Therapy evaluation		
3 Behavioural change and communication	Behavioural change and the RESET model	To introduce tools related to behavioural change and communication to promote responsible AMU.	Theory with interactive questions and individual assignments; Individual assignment on the RESET model; Peer feedback and discussion; 1 to 1.5 h online meeting with veterinary microbiologist and project leader.
	Communication		
4 Quantification of AMU	Quantification of systemic AMU in human and veterinary medicine	To introduce possible methods to quantify AMU.	Theory with interactive questions and individual assignments; Individual assignments on quantification of systemic and topical AMU within their own veterinary clinic; Peer feedback and discussion.
	Topical AMU	To introduce barriers and facilitators of quantification of AMU.	
		To promote optimization of registration of AMU within their own veterinary clinic.	
		To promote quantification and monitoring of AMU within their own veterinary clinic.	
		To gain insight in veterinary AMU within their own veterinary clinic, both quantitatively and qualitatively.	
5 How to continue?	How to apply Antimicrobial Stewardship within their own veterinary clinic?	To apply Antimicrobial Stewardship within their own veterinary clinic.	Video clip summarizing the whole course; Individual assignment to write down 5 SMART action points to work on in their own veterinary clinic; Peer feedback and discussion; 1 to 1.5 h online meeting with hospital pharmacist-clinical pharmacologist and project leader.

**Table 2:** Questions to evaluate achievement of the learning objectives of the online course. Questions were scored on a 6-point Likert scale (expressed in percentage and number of participants).

Questions	Totally Agree	Agree	Tend to Agree	Tend to Disagree	Disagree	Totally Disagree
- After participation in this online course, I am more aware of the risks of AMR and the importance of effective AMS.	17.5% (7)	67.5% (27)	15% (6)	0% (0)	0% (0)	0% (0)
- Because of participation in this online course, I have more insight into my own AM prescribing behaviour.	27.5% (11)	65% (26)	7.5% (3)	0% (0)	0% (0)	0% (0)
- Because of participation in this online course, I have a better understanding of what AMS comprises.	30% (12)	60% (24)	10% (4)	0% (0)	0% (0)	0% (0)
- Because of participation in this online course, I intend to use the Dutch Formulary and Guidelines on veterinary AMU more often.	42.5% (17)	32.5% (13)	25% (10)	0% (0)	0% (0)	0% (0)
- During participation in this online course, I received practical tools on behavioural change and communication, to use antimicrobials more responsibly.	12.5% (5)	60% (24)	22.5% (9)	5% (2)	0% (0)	0% (0)
- Because of participation in this online course, I have a better understanding of how to gain insight into AMU in the veterinary clinic I work in.	17.5% (7)	57.5% (23)	22.5% (9)	2.5% (1)	0% (0)	0% (0)
- Because of participation in this online course, responsible AMU receives more attention in the clinic I work in.	12.5% (5)	32.5% (13)	52.5% (21)	2.5% (1)	0% (0)	0% (0)
- Because of participation in this online course, I use AMs more responsibly.	35% (14)	47.5% (19)	17.5% (7)	0% (0)	0% (0)	0% (0)

## Data Availability

Not applicable.
